# Comparison of Magnetron-Sputtered and Cathodic Arc-Deposited Ti and Cr Thin Films on Stainless Steel for Bipolar Plates

**DOI:** 10.3390/ma17122864

**Published:** 2024-06-12

**Authors:** Nils Fredebeul-Beverungen, Maximilian Steinhorst, Teja Roch

**Affiliations:** Fraunhofer Institute for Material and Beam Technology IWS, 44145 Dortmund, Germany; maximilian.steinhorst@iws.fraunhofer.de (M.S.); teja.roch@iws.fraunhofer.de (T.R.)

**Keywords:** DC magnetron sputtering, cathodic arc evaporation, stainless steel bipolar plates, corrosion resistance, interfacial contact resistance

## Abstract

In this work, the potential of magnetron sputtering, as well as cathodic arc evaporation, is investigated with regard to its suitability as a bipolar plate coating of a PEM fuel cell. For this purpose, Cr and Ti thin films were deposited onto a 0.1 mm SS316L by varying the power and bias voltage. The surface structure and thickness of the coatings are examined via SEM and tactile profilometry. Moreover, the coating variants are compared with each other based on the electrical and electrochemical properties relevant to bipolar plates. The sputtered Cr thin films achieve the lowest contact resistance values and exhibit a columnar structure with a smooth surface. Regarding the electrochemical properties, titanium deposited via cathodic arc evaporation has a low current density in the passive region and high breakthrough potential. All in all, both deposition techniques have their individual advantages for the preparation of bipolar plates’ coatings. However, Ti thin films prepared via cathodic arc seem to be the most suitable option due to the combination of a high deposition rate, a low cost and good coating properties.

## 1. Introduction

As a clean, efficient and endlessly producible energy source, hydrogen offers great potential to save CO_2_ and protect the climate in the long term [1,2]. With fuel cells, the chemical energy of hydrogen can be converted directly into electrical energy and used to supply electricity. In principle, the only by-products in hydrogen fuel cells are water and heat. The resulting heat can, in turn, be used for heating. As no fossil fuel is burned, no air pollutants are produced, making hydrogen fuel cells an environmentally friendly alternative to fossil-based combustion engines [2,3,4]. One of the most promising fuel cells is the polymer electrolyte membrane (PEM) fuel cell [4,5,6,7,8]. It has already been tested in a wide variety of applications and is particularly popular in the mobile sector due to its simplicity, rapid operational readiness and dynamic operating behavior [4,5,6]. One of the main components of the PEMFC is the bipolar plate (BPP) [9]. It is responsible for electrical contacting, the gas supply, cooling and the removal of reaction water, for instance [5]. At the same time, it is in contact with the highly corrosive environment inside the PEMFC. Two of the most important properties of the BPP are, therefore, high corrosion resistance and good electrical electrical conductivity. Supplying a large market also requires a material that can be produced quickly and cheaply. Stainless steel is considered a suitable material due to its low cost, high strength, corrosion resistance and ease of processing [7,8,10]. However, in the highly corrosive environment of the fuel cell, there is a risk that the resulting corrosion products can poison the catalyst and reduce the efficiency of the cell [11,12]. For this reason, bipolar plates made of stainless steel are coated with corrosion-resistant and conductive thin films such as TiN, CrN or carbon to maintain a stable output during fuel cell operation [13,14,15,16,17]. These thin films are usually deposited using physical vapor deposition (PVD) [16]. One of the most widely used PVD processes is magnetron sputtering due to the high surface quality [18,19]. However, the degree of ionization is very low, and the deposition rate is limited. An alternative is cathodic arc evaporation, which is an established PVD method for the deposition of high-performance tribological coatings. It is characterized by a high degree of ionization and a high deposition rate [18,19]. However, the use of arc evaporation also has some disadvantages. When the target is melted, microparticles are ejected, which are deposited onto the substrate and incorporated into the growing coating. This can lead to growth defects and increased surface roughness, which can affect the quality and performance of the coating [18,19,20]. Previous works [21,22] compared both methods regarding their microstructure and mechanical properties. It was observed that the high adatom mobility of the arc evaporation process results in a denser structure and higher adhesive strength with the substrate. Additionally, increasing ion bombardment during the deposition of an evaporated coating causes an interruption to the growing columns and nucleation of new grains. While the sputter-deposited coating is rather homogeneous, the arc-evaporated coatings exhibit typical growth defects originating from incorporated microparticles. Both PVD methods exhibit advantages and drawbacks that may be advantageous or disadvantageous for coating metallic BPP. As can be ascertained from recent reviews regarding bipolar plate coatings [14,17,23], a direct comparison of magnetron sputtering and cathodic arc evaporation in the context of PEMFC, and here BPP-related properties, had not been conducted so far. Thus, the aim of this work was to investigate the potential of both processes with regard to their suitability for coating metallic bipolar plates for PEM fuel cells. For this purpose, Cr and Ti coatings were deposited on 0.1 mm-thick sheets of SS 316L using each process and varying the power and bias voltage. The resulting coatings were examined regarding their morphology and coating thickness and compared with each other on the basis of the electrical and electrochemical properties relevant to bipolar plates.

## 2. Materials and Methods

### 2.1. Sample Preparation

Chromium and titanium thin films were deposited on 0.1 mm-thick austenitic stainless steel 316L (1.4404 or X2CrNiMo17-12-2) sheets with a chemical composition (in wt %) of 0.02 C, 17.2 Cr, 10.1 Ni, 2.1 Mo, <2.0 Mn, <0.10 N and Fe. In addition to SS316L sheets (300 × 150 mm), 100Cr6 round specimens (d = 30 mm) were used for calotte grinding and fractions of silicon wafers (20 × 10 mm) for the investigation of fracture cross sections. All substrates were cleaned with isopropanol and dried with nitrogen before loading into the vacuum chamber. The vacuum chamber was evacuated to a base pressure below 3 × 10^−6^ mbar and preheated up to 200 °C for 15 min to prepare the chamber for deposition and create an environment that had as little contamination as possible. Ar gas (Linde) with a purity of 99.996% was fed into the chamber via mass flow controllers to set the working pressure. The temperature during deposition was maintained at 150 °C. Pure chromium with a purity of ≥99.5% and titanium grade 2 were used as targets. The deposition of the chromium and titanium thin films was carried out by means of cathodic arc evaporation and DC magnetron sputtering. Sample bias and sputter power, as well as the arc current, were varied for different sets of specimens. A summary of all the main deposition parameters is listed in Table 1. The different coating variants are named after the deposition method, coating material, arc current or sputtering power, and bias voltage. For example, MagCr-2.5-50 corresponds to a sputtered Cr coating using 2.5 kW of power and 50 V of bias voltage.

### 2.2. Morphology

The surface and cross-sectional morphology of the Cr and Ti thin films were analyzed using scanning electron microscopy (SEM, Phenom XL G2, Thermo Fisher Scientific, Waltham, MA, USA). Surface roughness was determined using 2D profilometry (Hommel Etamic T1000 Wave, Jenoptik, Villingen-Schwenningen, Germany). The coating thickness was measured using calotte grinding and SEM investigations on fracture cross sections.

### 2.3. Interfacial Contact Resistance

The contact resistance measurement was carried out at room temperature using Wang’s method [10]. The coated samples were rinsed with deionized water and dried with nitrogen before being sandwiched between two gas diffusion layers (GDL H14 by Freudenberg, Weinheim, Germany) and placed between two gold-plated copper contacts with a measurement area of 4 cm^2^. The sample, as well as the GDLs, was compressed with compaction forces between 25 and 200 N cm^−2^ using a tensile testing machine. The electrical resistance was determined by applying a current via the two copper contacts and measuring the voltage drop across the measurement path. The measured total resistance consisted of the sum of the bulk resistances and the contact resistances of the four contact pairs. The bulk resistances were very low compared to the contact resistances and can be neglected [24,25]. To eliminate the remaining contact resistances between the GDLs and the copper contacts, a reference measurement with one GDL between the contacts was conducted and subtracted from the sample measurement. The result was divided by two and multiplied by the measurement area of the copper contacts to obtain the interfacial contact resistance (mΩ cm^2^) between a GDL and the sample.

### 2.4. Electrochemical Characterization

The corrosion behavior of the coatings was investigated via potentiodynamic polarization tests after open-circuit stabilization for 1 h. The measurements were carried out at room temperature in a 0.5 M H_2_SO_4_ solution purged with Ar using a three-electrode test cell (Flatcell by IPS) with a platinum-coated titanium counter electrode and a Ag/AgCl reference electrode. The measurement area was 1 cm^2^. The polarization extended over a potential range from −0.64 V to 1.86 V vs. Ag/AgCl (equivalent to −0.5 to 2 V vs. SHE) at a scanning rate of 0.2 mV s^−1^. After the measurement, the recorded current was referenced to the standard hydrogen electrode (SHE), and the corrosion potential (E_*Corr*_), as well as the corrosion current density (I_*Corr*_), was determined. Both are commonly used as indicators of the corrosion resistance of a material [24,26].

## 3. Results and Discussion

### 3.1. Coating Thickness and Growth Rate

Table 2 and Table 3 list the determined coating thicknesses and growth rates of the coatings deposited via magnetron sputtering and arc evaporation, respectively. The growth rate was calculated from the ratio of the determined coating thickness to the corresponding coating time.

Overall, the thicknesses of the sputtered coatings ranged from 686 to 1945 nm, with growth rates of 11 to 32 nm min^−1^. As shown in Table 2, the Ti coatings had lower thicknesses compared to the Cr coatings under otherwise identical parameters. This was due to the lower sputter yield of Ti (0.6) compared to Cr (1.3) [27,28]. The sputter yield describes the ratio of outgoing target atoms to incoming ions. Regardless of the target material, the growth rate increases with increasing sputter power and decreases with increasing bias voltage on the substrate. Similar behavior was observed in the results of the coatings deposited via arc evaporation in Table 3. The thicknesses of the deposited coatings ranged from 110 to 915 nm with growth rates of 3 to 31 nm min^−1^. While the growth rate increased with an increasing arc current, it decreased with an increasing bias voltage. The bias voltage caused continuous ion bombardment of the substrate surface. By increasing the bias voltage, the energy of the accelerated ions was also increased, leading to the enhanced removal of the growing coating. This effect is known as “resputtering” [22,29]. In arc evaporation, Cr and Ti also exhibit different growth rates. In contrast to magnetron sputtering, significantly lower thicknesses were measured for the Cr coatings. This opposite behavior in terms of growth rates of the target materials can be attributed to the different physical processes involved in the transition of target atoms into the gas phase. In sputtering, atoms are dislodged from the target through collisions. The ratio of particle energy to the binding energy of the target atoms is crucial for the yield and, thus, the growth rate on the substrate. On the other hand, in arc evaporation, target atoms are released through the introduction of thermal energy. In addition to the binding energy of the target atoms, the melting temperature of the target material also plays a crucial role [18]. Higher melting materials like Cr exhibit higher-charged ions, combined with a lower particle current, resulting in a lower growth rate. When comparing the growth rates of the Cr coatings between the two methods, it is noticeable that the growth rates of the Cr coatings deposited via arc evaporation are significantly lower than those of the sputtered coatings. This is partly due to the high melting temperature of Cr. While this leads to a lower growth rate in arc evaporation, it has no influence on the physical effect of sputtering. Another possible explanation is the aforementioned effect of the removal of the growing coating. In magnetron sputtering, this is mainly done through the bombardment of Ar ions since the sputtered Cr atoms are electrically neutral and have only limited energy. In comparison, in arc evaporation, the removal occurs due to the almost complete ionization of the target atoms through the bombardment of Cr ions. These ions have higher energy and weight than Ar. The impact of Cr ions on the growing coating results in a more efficient impulse transfer [18] and an increased “resputtering” effect. The energy efficiency of the processes can be determined by considering the ratio of achieved growth rates to the powers used for the processes. Here, the choice of target material plays a significant role. While the sputter deposition was operated at powers of 2.5 and 5 kW, the power used in arc evaporation was approximately 1.5 and 2 kW. Despite the lower power, the deposition of Ti coatings via arc evaporation was significantly more efficient. On the other hand, the Cr coatings could be deposited slightly more efficiently via magnetron sputtering.

### 3.2. Coating Morphology

**Figure 1:**
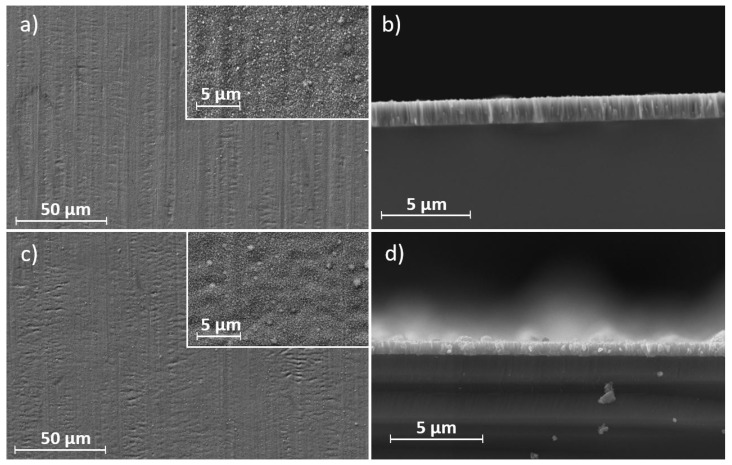
SEM images of the coated 316L sheets and Si-wafer cross sections: (**a**,**b**) Cr and (**c**,**d**) Ti deposited via magnetron sputtering.

To assess the coating structure and possible growth defects, the surfaces and cross sections of the deposited thin films were examined under the SEM. A comparison of SEM images of the sputtered Cr and Ti coatings is shown in Figure 1. As can be seen, the sputtered coatings of both target materials mostly have smooth surface structures.

**Figure 2:**
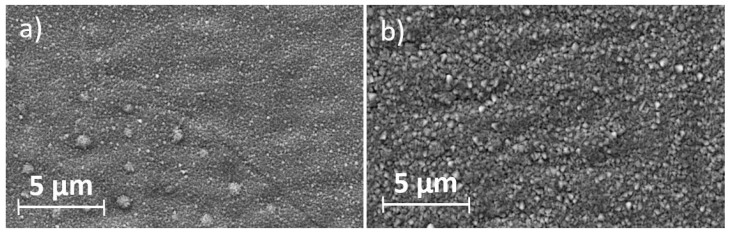
SEM images of sputtered Cr films at a sputtering power of (**a**) 2.5 kW and (**b**) 5 kW.

In addition to uniformly small grains, occasionally larger spherical protrusions (nodules) could be observed, which occurred more frequently in the Ti coatings. The cross sections exhibit a columnar structure, which can be attributed to Volmer–Weber island growth and a low homologous temperature [30]. When comparing the cross sections of the sputtered coatings, it is evident that the Cr coatings have finer and more pronounced columns compared to the Ti coatings. Figure 2 shows SEM images of sputtered coatings at different sputter powers. It can be observed that increasing the sputter power leads to an increase in grain size. In addition, the grains at a higher sputter power have a less rounded and increasingly sharp surface.

**Figure 3:**
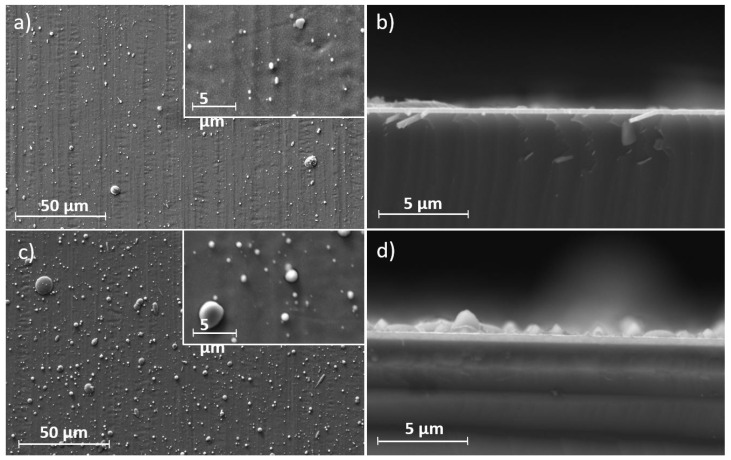
SEM images of the coated 316L sheets and the Si-wafer cross-sections: (**a**,**b**) Cr and (**c**,**d**) Ti deposited via arc evaporation.

A comparison of SEM images of the Cr and Ti thin films deposited via arc evaporation is shown in Figure 3. In contrast to the sputtered coatings, the coatings deposited via arc evaporation exhibit much rougher surfaces due to the emission of microparticles during evaporation. However, the cross sections have a denser structure without visible grains. Compared to the Cr coatings, the Ti coatings show a significantly higher density of microparticles. This can be explained by the different melting temperatures of the target materials. The greater the melting of the target material, the stronger the droplet emission [18,31]. Cr, with its higher melting temperature (1907 °C [32]) that is close to the boiling temperature, is less prone to the emission of microparticles compared to Ti (1668 °C [32]). Depending on the arc current used, an increase in the size and number of microparticles can be observed.

**Figure 4:**
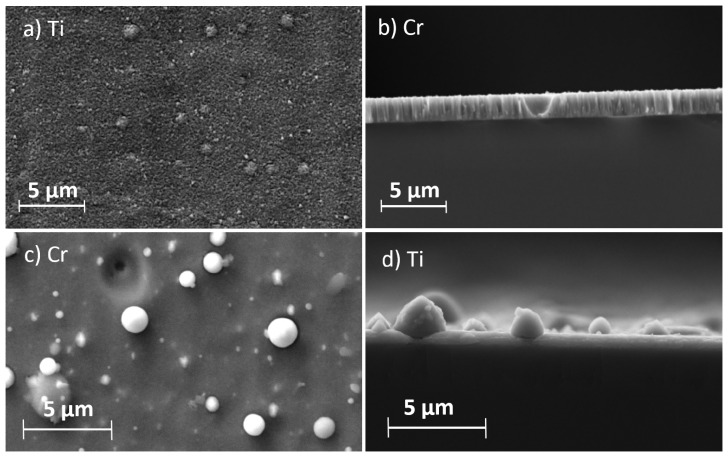
SEM images of coating defects occurring during coating via magnetron sputtering: (**a**) nodules; (**b**) craters. And coating defects occurring during coating via arc evaporation: (**c**) craters caused by removed microparticles; (**d**) coating growth around microparticles.

Figure 4 summarizes the defects and growth errors observed in each respective process. The coatings deposited via magnetron sputtering generally have fewer defects compared to the coatings deposited via arc evaporation. The most common defects in the sputtered coatings are nodules (see Figure 4a). Nodules are growth defects that can occur in all PVD processes [20]. Triggered by seed, they grow in the form of an inverted cone through the growing coating, depending on the rotation of the substrate and the angle of the impacting particles, resulting in bulges on the surface of the coating [20]. The seed can be small protrusions and irregularities on the substrate, as well as small foreign particles, dust, or target particles. Interruptions occur between nodules and the surrounding coating due to shadowing effects. These interruptions create weak points that can cause the nodule to detach from the coating and leave a crater behind (see Figure 4b). In arc evaporation, the majority of defects are caused by the emission of microparticles. At the point of impact, microparticles prevent further growth of the coating. Instead, the coating grows on and around the microparticles (see Figure 4d), partially incorporating them into the coating. When the microparticles detach from the coating, depressions and craters remain (see Figure 4c). If the microparticles are significantly smaller than the final coating thickness, they can also act as seeds for the formation of nodules. Thus, the presence of microparticles, under otherwise identical process conditions, increases the chance of nodule formation in arc evaporation compared to sputtering.

**Figure 5:**
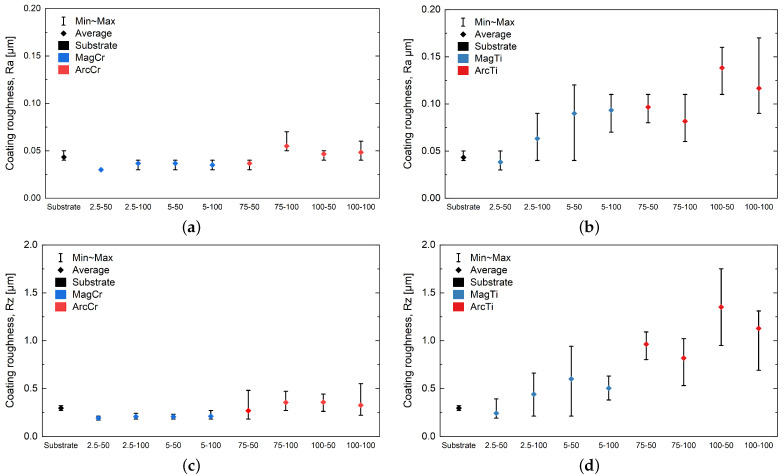
Surface roughness parameters (**a**,**b**) Ra and (**c**,**d**) Rz of the deposited Cr and Ti films on 316L sheets.

As was already observed in the SEM images, nodules and microparticles not only contribute to defects but also lead to increased surface roughness. This is confirmed by the roughness parameters shown in Figure 5. Due to the emission of microparticles, the coatings deposited via arc evaporation have higher surface roughness compared to the sputtered coatings. Depending on the target material and the arc current used, further increases in roughness can be observed. In contrast, an increase in bias voltage for the arc-evaporated Ti coatings resulted in a reduction in the roughness parameters. For the Cr coatings, no clear trend can be observed regarding the bias voltage.

Due to the smaller number of nodules, the sputtered Cr coatings have very low and uniform surface roughness values. The observed grain growth at a higher sputter power is reflected in increasing roughness parameters, but only for the Ti coatings (see Figure 5). In contrast to arc evaporation, an increase in applied bias voltage generally results in a slightly rougher surface.

### 3.3. ICR Measurement

**Figure 6:**
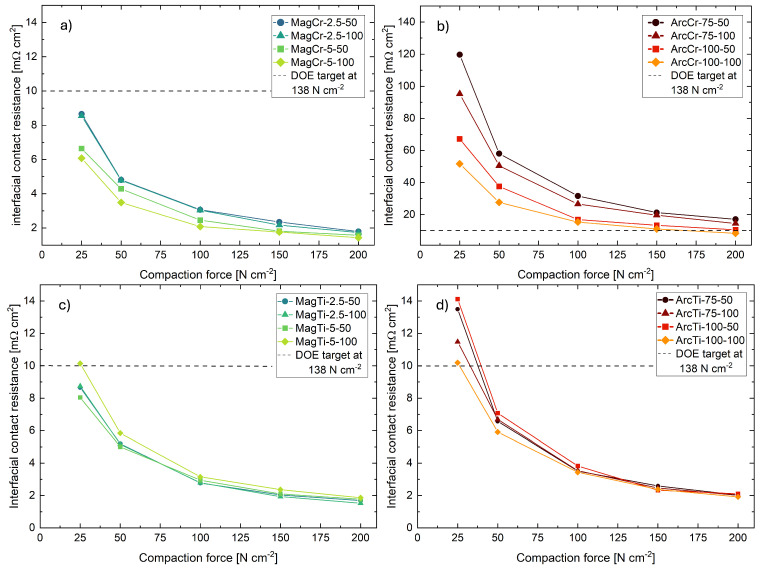
Measured contact resistances of the (**a**,**c**) magnetron-sputtered and (**b**,**d**) arc-evaporated Cr and Ti films.

The contact resistance between the bipolar plate and GDL has a strong influence on the total ohmic resistance of the PEMFC [5]; therefore, it is a crucial parameter for its performance. Increasing the compaction force leads to an enlargement of the contact area between the components and results in a reduction in the contact resistance. The contact resistance between the coatings and a GDL was determined using the setup described in Section 2.3 at compaction forces ranging from 25 to 200 N cm^−2^, and it took place shortly after the deposition. The measurement results are shown in Figure 6.

In general, the contact resistance of the uncoated 316L sheet, ranging from 400 to 60 mΩ cm^2^, could be improved with all deposited coatings across the entire measurement range. The sputtered coatings exhibit lower contact resistances compared to the coatings deposited via arc evaporation. While the contact resistances of all Ti coatings are relatively close to each other, the Cr coatings deposited via arc evaporation exhibit up to 10 times higher contact resistances than the sputtered Cr coatings. The ideal compaction force for PEMFC depends on the GDL used (type and manufacturer) and is usually represented as a range, rather than a specific value [33,34]. For bipolar plates in the mobile sector, the DOE (U.S. Department of Energy) has set a target value of 10 mΩ cm^2^ at a compaction force of 138 N cm^−2^ [35]. Except for the Cr coatings deposited via arc evaporation, all other samples are already below this target value, starting from 50 N cm^−2^. The Cr coatings deposited via arc evaporation only meet the DOE target at compaction forces above 138 N cm^−2^. At a compaction force of 150 N cm^−2^, the MagCr-5-50 sample achieves the lowest contact resistance of 1.81 mΩ cm^2^, while the ArcCr-75-50 sample has the highest contact resistance at 20.69 mΩ cm^2^. For the coatings deposited via arc evaporation, an increase in bias voltage at a constant arc current leads to a minor improvement in contact resistance. Compared to that, there is no clear effect of the bias voltage on the contact resistance of the sputtered coatings. Due to the otherwise identical conditions, the difference in contact resistances can be attributed to the formation of different coating structures and surface roughness resulting from the different deposition processes.

**Figure 7:**
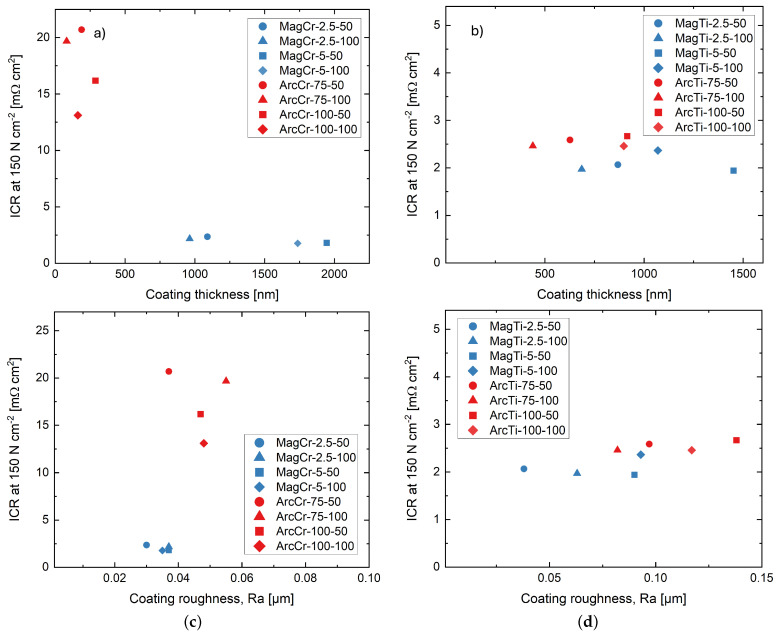
Contact resistance at a compaction force of 150 N cm^−2^ in relation to the (**a**,**b**) coating thickness and (**c**,**d**) surface roughness parameter Ra of the Cr and Ti films.

In Figure 7, the measured roughness parameter, R_*a*_, and layer thicknesses of the Cr and Ti coatings are plotted against the determined contact resistance at a compaction force of 150 N cm^−2^. Here, a tendency for the contact resistance to increase with higher R_*a*_ values can be observed. Additionally, it is noticeable that the contact resistances of the Ti coatings are all at a similar level, while there is a jump between the Cr coatings deposited via different processes. Regarding the coating thickness, no clear trend is evident. Surface roughness is a well-known factor influencing contact resistance, according to the literature [34,36,37,38,39]. Studies have shown that surface roughness can be a significant factor in minimizing contact resistance, depending on the specific GDL used, the mechanical properties of its fibers, and the applied compaction force. These factors determine the extent to which the GDL can adapt to the roughness of the contacting surface [38]. The better the GDL can adapt to the roughness, the larger the contact area, and the lower the resistance can be. If the roughness is too high or not sufficiently large, it can lead to an increase in contact resistance. If this is taken into account with regard to the different contact resistances of the two deposition processes, it is possible that the microparticles formed during arc evaporation, due to their localized significant height difference, may result in less contact between the GDL and the sample, thereby increasing the contact resistance. For the Ti coatings, the difference in both R_*a*_ and R_*z*_ values is small, which leads to similar contact resistances. On the other hand, for the Cr coatings, the R_*a*_ values are close to each other, but the R_*z*_ values differ significantly. This is due to the relatively low number of microparticles. When calculating R_*a*_, these microparticles do not have as much influence. However, R_*z*_ refers to the largest deviations within the sampling length, which, in this case, are represented by the microparticles.

### 3.4. Electrochemical Characteristics

The interior of a PEMFC represents a highly corrosive environment. The corrosion behavior of the bipolar plates plays a crucial role in the longevity and performance of the cell. Polarization curves are used to investigate the corrosion behavior of the deposited coatings. These curves provide information about the corrosion potential and corrosion current density, which can be used as indicators of the corrosion resistance of a material. A high (noble) corrosion potential and a low corrosion current density indicate high corrosion resistance [24]. The typical potential of a bipolar plate in contact with the electrode is 0.1 V vs. SHE on the anode side and 0.8 V vs. SHE on the cathode side [10,24]. At these potentials, the measured current density should be as low as possible [35,40,41]. The DOE recommends a current density of <1 μA cm^−2^ without an anodic peak during passivation [35].

**Figure 8:**
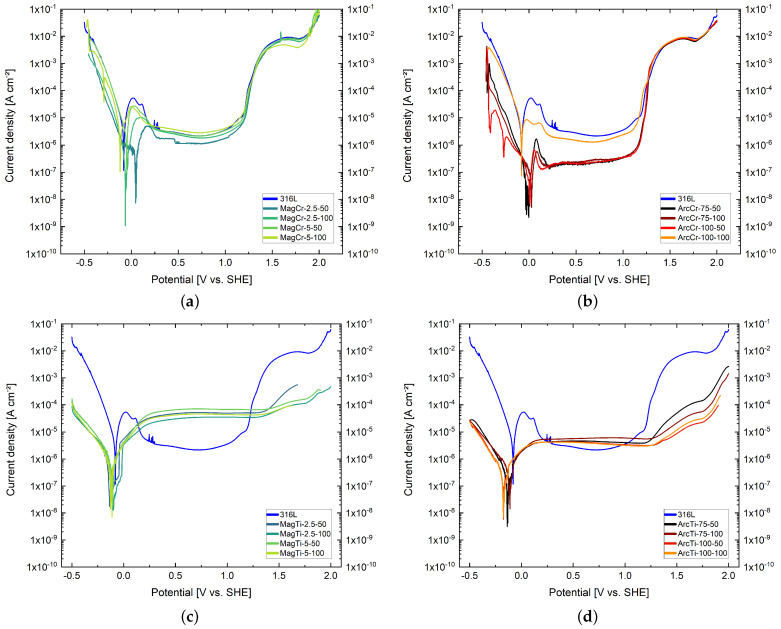
Potentiodynamic polarization curves of the 316L substrate and the (**a**,**c**) magnetron-sputtered and (**b**,**d**) arc-evaporated Cr and Ti films in 0.5 M H_2_SO_4_ at room temperature.

The 316L substrate reaches its corrosion potential at −0.08 V vs. SHE and is characterized by an anodic peak with a critical current density of 40 μA cm^−2^, followed by a sharp increase in current density after reaching the breakdown potential at 1.3 V vs. SHE (see Figure 8). The breakdown potential marks the potential at which, after passivation, a sharp increase in current density is observed, indicating enhanced metal dissolution. At this point, the formed passive film becomes unstable and begins to dissolve. The passive state extends over a range of 0.2–1.2 V vs. SHE with a passive current density of approximately 2 μA cm^−2^.

The measured polarization curves of the deposited Cr coatings are shown in Figure 8a,b. The Cr coatings deposited via magnetron sputtering generally show a similar trend to the substrate but with a slightly improved corrosion resistance and, in some cases, a significantly reduced critical current density. Overall, the MagCr-2.5-50 coating achieved the best results, including a lower current density in the passive state. However, the passive state itself still extended over the same potential range, and no effect on the breakdown potential is evident. The coatings deposited with a higher sputter power and bias voltage gradually approached the corrosion behavior of the stainless steel substrate, thus deteriorating corrosion resistance.

Comparable to the sputtered coatings, the Cr coatings deposited via arc evaporation exhibit a significantly reduced critical current density, which can be seen in Figure 8b. However, unlike the sputtered coatings, all the arc-evaporated coatings show a lowering of the passive state. This effect is particularly pronounced for the ArcCr-75-50, ArcCr-75-100, and ArcCr-100-50 coatings, whose passive current density of approximately 0.2 μA cm^−2^ is 10 times lower compared to the substrate. Furthermore, except for the ArcCr-100-100 coating, all the coatings show an increase in corrosion potential and, thus, improved corrosion resistance. In the case of ArcCr-100-50, more than one corrosion potential can be observed. This is an electrochemical phenomenon that can occur in pure Cr in vented sulfuric acid and prior exposure to air due to different reaction mechanisms during hydrogen evolution [42,43]. It should be noted that the occurrence of multiple corrosion potentials is often considered a risk in practice, as the sample theoretically can start corroding at any of these potentials [26].

The measured polarization curves of the deposited Ti coatings are shown in Figure 8c,d. The Ti coatings exhibit similar trends for both deposition methods. In both cases, both the corrosion potential and corrosion current density have decreased. The achieved corrosion potentials of the Ti coatings are between that of pure Ti and the substrate. The passive current density of the sputtered Ti coatings is significantly higher than that of the arc-evaporated coatings. Unlike the Cr coatings, the Ti coatings do not exhibit an anodic peak during passivation. Additionally, the passive state extends over a larger potential range compared to the Cr coatings. This is partly because of the breakdown potential of the Ti coatings, which is located at a higher potential.

After the measurements, a complete detachment or dissolution of all Cr coatings could be observed, regardless of the deposition process. The electrolyte in the measurement cell was yellowish and discolored. In addition, small greenish particles remained on the measurement surface of the sample. The yellow coloration of the electrolyte can be attributed to the formation of Cr^*VI*^ [44]. However, for some of the sputtered Cr coatings, a bluish, discolored electrolyte could also be observed after the end of the measurement, which may have been caused by different oxidation states [45], resulting in different corrosion products and remnants of the detached Cr coating. The Ti coatings remained largely intact due to the higher breakdown potential, even after the polarization measurements; thus, no discoloration of the electrolyte could be observed. This corrosion behavior is more suitable in the initial phase of the PEMFC, when cathodic potentials of up to 1.4 V can occur [40]. In the case of the Ti coatings, this value is close to the breakdown potential. Regarding the Cr coatings and the substrate, both are already in the transpassive state at this potential. Here, the passive layer begins to dissolve. The detached material and released metal ions can have a negative impact on the MEA in practical applications and reduce the performance and lifespan of the cell [11,12].

Overall, the coatings deposited via arc evaporation exhibited more beneficial corrosion for use in the fuel cell environment. While the sputtered coatings may have higher corrosion potential, the coatings deposited via arc evaporation have significantly lower corrosion current densities. The current densities at potentials of 0.1 and 0.8 V vs. SHE, which are relevant for the fuel cell, were lower in all cases compared to the sputtered coatings, and they were also much closer to the value of 1 μA cm^−2^ recommended by the DOE. This is particularly advantageous, considering the elevated temperatures inside a fuel cell, which intensifies the corrosive attack.

## 4. Conclusions

In this work, the potential of the industrially widespread PVD methods, arc evaporation and magnetron sputtering, for coating metallic bipolar plates was investigated. Cr and Ti coatings were deposited using both methods, and variations in power and bias voltage were applied. The resulting coatings were examined in terms of their thickness, structure, electrical, and electrochemical properties. The main conclusions are as follows:

1. Based on the measured coating thicknesses, the growth rate of the coatings was determined. Both methods showed a decrease in the growth rate with an increasing bias voltage due to resputtering effects. The strength of this effect depended on the combination of the method and target material used. Thus, the efficiency of the methods showed a dependence on the target material.
2. In the SEM investigations, it could be observered that the use of magnetron sputtering resulted in columnar structured coatings with mostly smooth surfaces but with occasional nodes. In contrast, arc evaporation resulted in denser coatings with a large number of microparticles, leading to increased surface roughness and growth defects. Moreover, the Cr coatings exhibited fewer and smaller microparticles, which can be attributed to the higher melting point.
3. The contact resistance of the 316L substrate could be improved with all coatings. The sputtered coatings showed lower contact resistances than the arc-evaporated coatings, regardless of the target material used. The contact resistance of 10 mΩ cm^2^ recommended by the DOE was already exceeded in almost all samples at a contact pressure of 50 N cm^−2^. The sputtered Cr coatings achieved the best contact resistance.
4. The arc-evaporated coatings showed better corrosion behavior compared to the magnetron-sputtered coatings. In particular, the arc-evaporated Ti coatings have beneficial electrochemical properties for use as bipolar plate coating, with a low passivation current density without an active peak, a stable passive region, and a high breakdown potential.

In conclusion, this work has shown that the magnetron-sputtering and arc-evaporation methods have individual advantages for the coating of metallic bipolar plates. While sputtered coatings achieve lower contact resistances, arc-evaporated coatings exhibit more suitable corrosion behavior. However, in the case of bipolar plates, the goal is to combine both properties. Therefore, further investigations are planned to investigate the influence of gas pressure and the process temperature.

## Figures and Tables

**Table 1:** Deposition parameters used for cathodic arc evaporation and magnetron sputtering.

Deposition Parameters	Cathodic Arc Evaporation	Magnetron Sputtering
Working pressure	1 × 10^−4^ mbar	4 × 10^−3^ mbar
Process temperature	150 °C	150 °C
Ar flow	10 sccm	52 sccm
Coating time	30 min	60 min
DC power	-	2.5 kW, 5 kW ^1^
Arc current	75 A, 100 A	-
Bias voltage	50 V, 100 V	50 V, 100 V
Target to substrate distance	200 mm	200 mm
Rotational speed	5 rot./min	5 rot./min

^1^ 5 W cm^−2^, 10 W cm^−2^.

**Table 2:** Coating thickness and growth rate of the sputtered coatings.

Target Material	Sputtering Power [kW]	Bias Voltage [V]	Coating Thickness [nm]	Growth Rate [nm min^−1^]
Cr	2.5	50	1090	18
		100	963	16
	5	50	1945	32
		100	1737	29
Ti	2.5	50	868	14
		100	686	11
	5	50	1452	24
		100	1070	18

**Table 3:** Coating thickness and growth rate of the arc-evaporated coatings.

Target Material	Arc Current [A]	Bias Voltage [V]	Coating Thickness [nm]	Growth Rate [nm min^−1^]
Cr	75	50	189	6
		100	110	3
	100	50	288	10
		100	162	5
Ti	75	50	627	21
		100	439	15
	100	50	915	31
		100	898	30

## Data Availability

The study’s data are contained within the article.

## References

[1] Heinzel A. (2009). Stand der Technik von Polymer-Elektrolyt-Membran-Brennstoffzellen—Ein Überblick. Chemie Ingenieure Technik 81.

[2] Abe J.O., Popoola A.P.I., Ajenifuja E., Popoola O.M. (2019). Hydrogen energy, economy and storage: Review and recommendation. Int. J. Hydrogen Energy.

[3] Kendall K., Ye S., Liu Z. (2023). The Hydrogen Fuel Cell Battery: Replacing the Combustion Engine in Heavy Vehicles. Engineering.

[4] Kurzweil P., Schmid O. (2003). Brennstoffzellentechnik: Grundlagen, Materialien, Anwendung, Gaserzeugung.

[5] Göbel M. (2021). Untersuchung des Einflusses der Gasdiffusionslage auf das Wassermanagement in der Automobilen PEM-Brennstoffzelle. Ph.D. Thesis.

[6] Sammes N. (2006). Fuel Cell Technology: Reaching towards Commercialization.

[7] Tawfik H., Hung Y., Mahajan D. (2007). Metal Bipolar Plates for PEM Fuel Cell—A Review. J. Power Sources.

[8] Xu Z., Qiu D., Yi P., Peng L., Lai X. (2020). Towards Mass Applications: A Review on the Challenges and Developments in Metallic Bipolar Plates for PEMFC. Prog. Nat. Sci. Mater. Int..

[9] Heinzel A. (2009). Reviewing Metallic PEMFC Bipolar Plates. Fuel Cells.

[10] Wang H., Sweikart M., Turner J. (2003). Stainless steel as bipolar plate material for polymer electrolyte membrane fuel cells. J. Power Sources.

[11] Mittal V.O., Kunz H.R., Fenton J.M. (2007). Membrane Degradation Mechanisms in PEMFCs. J. Electrochem. Soc..

[12] Papadias D., Ahluwalia R., Thomson J., Meyer H., Brady M., Wang H., Turner J., Mukundan R., Borup R. (2015). Degradation of SS316L bipolar plates in simulated fuel cell environment: Corrosion rate, barrier film formation kinetics and contact resistance. J. Power Sources.

[13] Hamilton P. (2013). The Development of PVD Coatings for PEM Fuel Cell Bipolar Plates. Ph.D. Thesis.

[14] Liu R., Jia Q., Zhang B., Lai Z., Chen L. (2022). Protective Coatings for Metal Bipolar Plates of Fuel Cells: A Review. Int. J. Hydrogen Energy.

[15] Steinhorst M., Giorgio M., Roch T., Leyens C. (2023). Bias Voltage Dependency of Structural and Bipolar Plate-Related Properties of Cathodic Arc-Deposited Carbon-Based Coatings. ACS Appl. Mater. Interfaces.

[16] Wu S., Yang W., Yan H., Zuo X., Cao Z., Li H., Shi M., Chen H. (2021). A review of modified metal bipolar plates for proton exchange membrane fuel cells. Int. J. Hydrogen Energy.

[17] Gao X., Chen J., Xu R., Zhen Z., Zeng X., Cui X.C.L. (2024). Research progress and prospect of the materials of bipolar plates for proton exchange membrane fuel cells (PEMFCs). Int. J. Hydrogen Energy.

[18] Schultrich B. (2016). Dünnschichttechnologie. VIP Journal.

[19] Gudmundsson J.T., Anders A., Von Keudell A. (2022). Foundations of Physical Vapor Deposition with Plasma Assistance. Plasma Sources Sci. Technol..

[20] Panjan P., Drnovšek A., Gselman P., Čekada M., Panjan M. (2020). Review of Growth Defects in Thin Films Prepared by PVD Techniques. Coatings.

[21] Chen L., Chang K.K., Du Y., Li J.R., Wu M.J. (2011). A comparative research on magnetron sputtering and arc evaporation deposition of Ti–Al–N coatings. Thin Solid Films.

[22] Tritremmel C. (2007). Comparison of Magnetron Sputtering and Arc Evaporation by Al-Cr-N Hard Coatings. Master’s Thesis.

[23] Bohackova T., Ludvik J., Kouril M. (2021). Metallic Material Selection and Prospective Surface Treatments for Proton Exchange Membrane Fuel Cell Bipolar Plates—A Review. Materials.

[24] Yoon W., Huang X., Fazzino P. (2008). Evaluation of coated metallic bipolar plates for polymer electrolyte membrane fuel cells. J. Power Sources.

[25] Jin J., Zhu Z., Zheng D. (2017). Influence of Ti content on the corrosion properties and contact resistance of CrTiN coating in simulated proton exchange membrane fuel cells. Int. J. Hydrogen Energy.

[26] Liening E. (1986). Process Industries Corrosion—Electrochemical Corrosion Testing Techniques.

[27] Sciences A. (2023). Sputtering Yields.

[28] Savvides N., Knittel A. (2004). Ion Beam Figuring of Optics.

[29] Gregoire J., Lobovsky M., Heinz M. (2007). Resputtering phenomena and determination of composition in codeposition. Phys. Rev. B.

[30] Alfonso E., Olaya J., Cubillos G. (2012). Thin Film Growth through Sputtering Technique and Its Applications.

[31] Peng B., Xu Y., Du J. (2022). Influence if preliminary metal-ion etching on the topography and mechanical behavior of TiAlN coatings on cemented carbides. Surf. Coat. Technol..

[32] Askeland D., Fulay P., Wright W. (2010). The Science and Engineering of Materials.

[33] Irmscher P. (2019). Mechanische Eigenschaften von Polymer-Elektrolyt-Membran-Brennstoffzellen.

[34] Atyabi S., Afshari E., Wongwises S. (2019). Effects of assembly pressure on PEM fuel cell performance by taking into accounts electrical and thermal contact resistances. Energy.

[35] U.S. Department of Energy (2017). Hydrogen and Fuel Cell Technologies Office Multi-Year Research, Development, and Demonstration Plan.

[36] Liskiewicz T., Kubiak K., Mann D. (2020). Analysis of surface roughness morphology with TRIZ methodology in automotive electrical contacts: Design against third body fretting-corrosion. Tribol. Int..

[37] Avasarala B., Haldar P. (2009). Effect of surface roughness of composite bipolar plates on the contact resistance of a proton exchange membrane fuel cell. J. Power Sources.

[38] Kraytsberg A., Auinat M., Ein-Eli Y. (2007). Reduced contact resistance of PEM fuel cell’s bipolar plates. J. Power Sources.

[39] Liang H., Xu J., Zhou D. (2016). Thickness dependent microstructureal and electrical properties of TiN thin films prepared by DC reactive Magnetron sputtering. Ceram. Int..

[40] Nikolov K., Bunk K., Jung A., Gerlach J.W., Kaestner P., Klages C.-P. (2017). Combined plasma surface modification of austenitic stainless steel for bipolar plates. Surf. Coat. Technol..

[41] Rajasekar S., Chetty R., Neelakantan L. (2015). Low-nickel austenitic stainless steel as an alternative to 316L bipolar plate for proton exchange membrane fuel cells. Surf. Coat. Technol..

[42] Popić J., Dražić D. (2004). Electrochemistry of active chromium: Part II. Three hydrogen evolution reactions on chromium in sufuric acid. Electrochim. Acta.

[43] Wilde B., Hodge F. (1969). The cathodic discharge of hydrogen on active and passive chromium surfaces in dilute sulphuric acid solutions. Electrochim. Acta.

[44] Guertin J., Jacobs J.A., Avakian C.P. (2005). Chromium (VI) Handbook.

[45] Abdallah M. (2003). Corrosion behaviour of 304 stainless steel in sulphuric acid solutions and its inhibition by some substituted pyrazolones. Mater. Chem. Phys..

